# Bringing together emerging and endemic zoonoses surveillance: shared challenges and a common solution

**DOI:** 10.1098/rstb.2011.0362

**Published:** 2012-10-19

**Authors:** Jo Halliday, Chris Daborn, Harriet Auty, Zacharia Mtema, Tiziana Lembo, Barend M. deC. Bronsvoort, Ian Handel, Darryn Knobel, Katie Hampson, Sarah Cleaveland

**Affiliations:** 1 Boyd Orr Centre for Population and Ecosystem Health, College of Medicine, Veterinary Medicine and Life Sciences, University of Glasgow, Glasgow G12 8QQ, UK; 2 Tropical Vet Services, Tanzania; 3 Ifakara Health Institute, PO Box 78373, Kiko Avenue, Mikocheni, Dar es Salaam, Tanzania; 4 The Roslin Institute and Royal (Dick) School of Veterinary Studies, University of Edinburgh, Easter Bush, Midlothian EH25 9RG, UK; 5 Department of Veterinary Tropical Diseases, Faculty of Veterinary Science, University of Pretoria, Onderstepoort 0110, South Africa

**Keywords:** surveillance, zoonoses, emerging diseases, neglected diseases, diagnostic capacity, information technologies

## Abstract

Early detection of disease outbreaks in human and animal populations is crucial to the effective surveillance of emerging infectious diseases. However, there are marked geographical disparities in capacity for early detection of outbreaks, which limit the effectiveness of global surveillance strategies. Linking surveillance approaches for emerging and neglected endemic zoonoses, with a renewed focus on existing disease problems in developing countries, has the potential to overcome several limitations and to achieve additional health benefits. Poor reporting is a major constraint to the surveillance of both emerging and endemic zoonoses, and several important barriers to reporting can be identified: (i) a lack of tangible benefits when reports are made; (ii) a lack of capacity to enforce regulations; (iii) poor communication among communities, institutions and sectors; and (iv) complexities of the international regulatory environment. Redirecting surveillance efforts to focus on endemic zoonoses in developing countries offers a pragmatic approach that overcomes some of these barriers and provides support in regions where surveillance capacity is currently weakest. In addition, this approach addresses immediate health and development problems, and provides an equitable and sustainable mechanism for building the culture of surveillance and the core capacities that are needed for all zoonotic pathogens, including emerging disease threats.

## 1. Introduction

Zoonotic pathogens predominate as the cause of both novel and re-emerging infectious diseases [1]. As a result, the question of how to conduct surveillance for zoonotic diseases at the global scale has been prioritized. The importance of interactions between human, wildlife and domestic animal populations, the potential for the rapid global spread of emerging pathogens and appreciation of the need to carry out surveillance for as yet unknown pathogens demonstrate the need for new approaches to surveillance that are both more comprehensive and more flexible than those that have existed previously [2]. At the same time, there are concerns about the impacts of neglected, endemic zoonotic pathogens, particularly in marginalized and impoverished communities, and the critical role of surveillance in generating data to demonstrate the true burden of these diseases for public health priority-setting [3–5].

Effective integration of surveillance in both human and animal populations is widely recognized as the key to the successful surveillance of emerging diseases [2,6,7], but a review of articles published between 1992 and 2006 indicated that only 19 per cent of studies relevant to surveillance systems for emerging diseases included evaluation of both human and animal data [8]. Considerable investments have been made in recent years to address these deficits through initiatives that incorporate zoonoses specifically within international surveillance systems for infectious diseases and include surveillance of both human and animal populations. Examples include international, multi-agency systems for early detection of disease outbreaks such as the Global Early Warning and Response System (GLEWS) [9] and the Global Outbreak Alert and Response Network (GOARN) [10], which bring together existing institutions such as the Food and Agriculture Organization of the United Nations (FAO), World Organisation for Animal Health (OIE), World Health Organization (WHO) and others to share information, pool resources and coordinate efforts to detect and respond to disease outbreaks. ProMED uses in-country infectious disease experts to validate reports and provides a model for an affordable web-based system that may be suitable for resource-poor countries [11,12]. Several international systems and programmes for animal and human health have also been designed to promote capacity building at the national level, such as the International Health Regulations (IHR) [13], the Global Framework for the progressive control of Transboundary Animal Diseases (GF-TADS) [14] and the Global Disease Detection Programme of the Centers for Disease Control and Prevention [15].

Early detection and reporting of disease cases is critical for initiating preventive measures before localized outbreaks develop into large-scale epidemics. By definition, zoonoses originate in animal hosts, and surveillance of animal populations offers the opportunity to detect pathogens earlier in the transmission or emergence pathway, before introduction to, and potential spread in, human populations. This is currently most feasible for known zoonoses where animal cases precede human infections (e.g. Ebola in great apes [16], West Nile virus in crows [17–19], Rift Valley fever in livestock [20] and highly pathogenic avian influenza (HPAI) in susceptible bird species [21,22]). In these cases, the observation of animal cases can be used to trigger targeted surveillance for high-risk human populations to improve the chances of early detection and prevention.

Using a database of all disease outbreaks reported to WHO from 1996 to 2009, Chan *et al*. [23] attempted to quantify global surveillance capacities for detecting and communicating disease outbreaks. Their findings showed that in many regions outbreak detection and reporting occurs very rapidly and that the intervals between the start of an outbreak, its detection and public communication had generally decreased over time. Considering all regions globally in 2009, the median delays to detection and communication were just 13.5 and 19 days, respectively, but with considerable geographical variation [23]. Of all outbreaks considered, 53 per cent were reported from Africa, where both detection and public communication delays were longest and several delays of over 150 days were observed [23]. Spatial reporting biases have also been identified for emerging infectious diseases specifically, with reduced reporting of disease events from developing regions [24]. Because of the potential for rapid international spread of infectious diseases, this reduced capacity for early detection of disease outbreaks in many developing regions has implications for the global community as a whole.

Despite increased interest and investment in global surveillance, the scale of the task is considerable and many challenges remain. Cutting-edge research to identify predictors of disease emergence and technological innovations for pathogen screening and discovery are promising advances that may help focus future surveillance efforts, but these approaches are currently associated with uncertainties that limit their effective translation (discussed in §2*a*). Much investment at the international and national levels has addressed technological limitations, such as laboratory diagnostic facilities and communications infrastructures. While these are clearly essential, there is also a need to recognize constraints that operate at the grassroots level; animal and human health workers who work at the community level in developed and developing countries are the primary source of surveillance data, and it is important to understand the factors that affect their ability and willingness to report disease outbreaks.

Building global systems for zoonoses surveillance involves a wide range of stakeholders, each with different perspectives and priorities. A key consideration is that the relative costs and benefits of emerging disease surveillance differ among high- and low-income countries. Establishing surveillance systems to prevent potential pandemic spread of emerging zoonoses will benefit both high- and low-income countries. However, emerging disease surveillance also carries high costs, some of which disproportionately affect developing countries, and rarely addresses the disease problems that already pose a far greater burden for impoverished communities. These include, for example, the endemic and neglected zoonoses such as rabies, anthrax, bovine tuberculosis, brucellosis, leptospirosis, and a range of helminth and protozoal infections [4,5].

Emerging and endemic zoonoses share many common characteristics that could be exploited in combined surveillance approaches to address zoonotic diseases as a whole and provide benefits for all global partners. In this paper, we discuss the surveillance of zoonoses with a focus on the perspectives of developing countries. We describe some of the important barriers that obstruct the reporting of zoonoses, and suggest pragmatic approaches that have potential to enhance surveillance of both emerging and endemic zoonoses with global and equitable benefits.

## 2. Zoonoses and disease reporting

**Figure 1.**
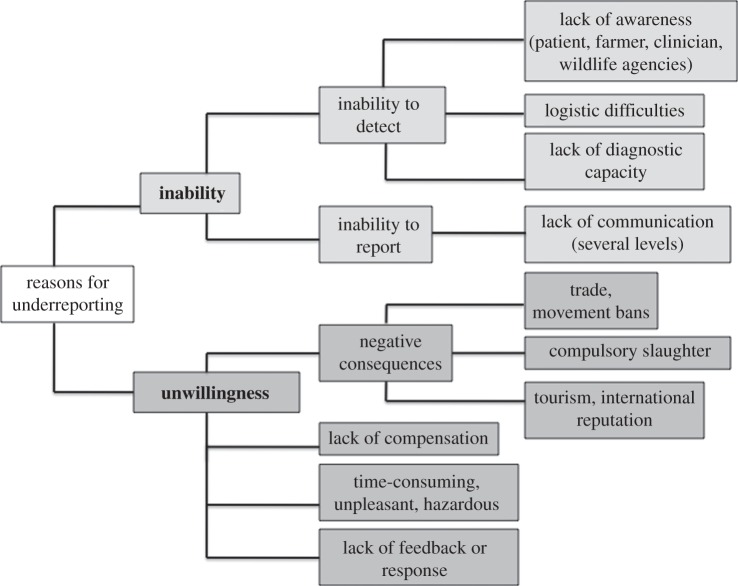
Scheme outlining reasons for the underreporting of zoonotic diseases. Adapted from World Bank [7].

**Figure 2.**
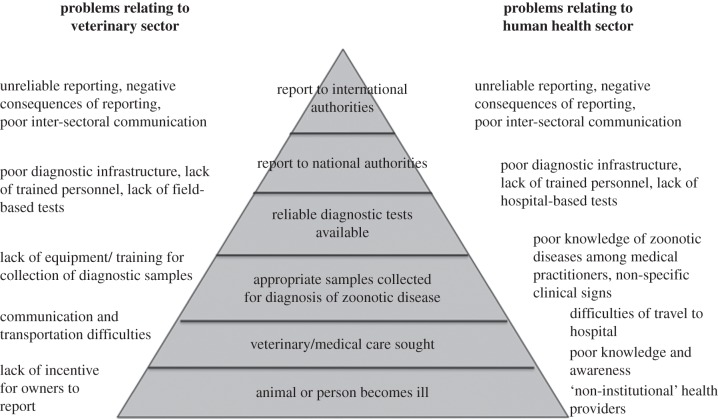
Factors contributing to the underreporting of zoonotic disease within the human and animal health sectors. The quality and quantity of surveillance data deteriorate at each step in this hierarchy, resulting in limited capacity to inform appropriate, timely and effective responses to disease outbreaks.

Zoonoses are often underreported, and it is important to understand and tackle the reasons for this [5]. Many factors contribute to underreporting, arising from both an inability and an unwillingness to report (figures 1 and 2). The relative importance of these factors varies in different situations, but they often act in combination to stifle the collection and distribution of accurate and comprehensive data, particularly in resource-poor settings.

### (a) Application of novel technologies: are these helping to address gaps in surveillance capacity?

Increasingly, surveillance systems are incorporating new technologies and technical approaches [11,25–27]. These relate both to the techniques used for collection and communication of data, and to the data-sources that are used. Internet-based systems provide powerful new tools for real-time reporting and communication of surveillance data. Several web-based systems, such as ProMED [12], the Global Public Health Intelligence Network (GPHIN) [28], HealthMap [29] and BioCaster [30], are now established. Many of these systems rely on sophisticated software to search and extract information from web-based sources and are motivated by a need to make better use of currently existing data. While frequently designed to achieve global coverage, internet-based systems do not necessarily address the geographical gaps in surveillance capacity, and remain limited by fundamental deficiencies in communications infrastructure in less-developed regions. For example, analysis of data obtained through the HealthMap system found a bias towards increased reporting from countries with more media outlets, more developed public health resources and greater electronic communications infrastructure [25]. The reliance of high-tech surveillance systems upon existing communications infrastructures such as the internet, and, in some cases, the high costs of accessing data (e.g. GPHIN) have the potential to compound the degree to which developing countries are underrepresented, with negative repercussions for national surveillance capacity and investment in disease control.

Efforts have been made to identify factors associated with disease emergence so that surveillance resources can be focused where emergence is most likely [1,24,31–33]. While progress has been made in analysing the geographical distribution of emerging disease events to identify emergence ‘hotspots’ [24], the data informing these models are currently limited. As a result, their predictive power, while seductive in terms of providing a focus for targeting resources, remains uncertain. It is clear that developing surveillance strategies that encompass a global geographical span poses a major challenge. However, potential approaches to the problem can be identified, including a focus on enhancing capacity in those areas where it is currently weakest.

Technological advances in laboratory techniques for pathogen screening and discovery also offer considerable potential for detecting novel micro-organisms in animal hosts, including potential zoonoses that are not yet known to be human or animal pathogens [34]. However, our ability to interpret these data, to assess the possible transmissibility and pathogenicity of micro-organisms and to predict disease emergence is currently limited. Many uncertainties remain as to the appropriate response to the detection of numerous novel micro-organisms in human or animal populations.

Mobile phone technology has enormous potential for improving health systems, including surveillance. Massive increases in network coverage, handset ownership and therefore familiarity with the technology mean that mobile phones hold particular promise in areas that are currently least well served by existing systems. Several of the more developed mobile-phone-based participatory systems for public health are described by Freifeld *et al*. [26], and an inventory of mobile phone data collection applications (the majority of which are implemented in Africa) is maintained online (http://www.unglobalpulse.org/resources/mobile-phone-data-collection-inventory). Most of these applications focus on human populations, and their potential application for animal disease surveillance has yet to be fully explored or exploited [35].

While providing many potential advantages, several constraints of mobile-phone-based surveillance can be identified. The major expenses associated with establishing and running mobile-phone-based systems are associated with the hardware required [35]. Many surveillance applications that are currently in development rely on expensive smartphones, which are not yet widely available in resource-poor settings. Although the costs of these smartphones are anticipated to fall rapidly [26], there is an argument for designing systems that make better use of the mobile phone technologies that are already most widely distributed, allowing for more rapid practical application. Open source software packages such as EpiSurveyor, RapidSMS, Java Rosa, FrontlineSMS and Nokia Data Gathering are all now widely used, and technical support for in-country partners is increasingly available, for example through the Open Mobile Consortium (OMC, http://www.open-mobile.org/). Another major consideration is that the current proliferation of mobile-phone-based systems has the potential to overwhelm rather than to assist the existing surveillance networks. Care needs to be taken to ensure that integrated and efficient systems are established to meet the needs of stakeholders rather that generating a suite of parallel reporting systems each with their own focus and different hardware and software requirements.

One of the greatest strengths of surveillance systems that use mobile phones is the capacity for two-way transfer of information, and the value of feedback in reporting systems is discussed later. Fundamentally though, mobile phones remain a communication tool and unless sufficient investments are made in the grassroots surveillance workers who use these tools, and in overcoming the barriers to reporting (figures 1 and 2), mobile-phone-based systems are likely to suffer from many of the same challenges as paper-based systems.

### (b) International reporting regulations

Zoonoses often fall in the gap between the animal and human health sectors and this can lead to an underinvestment in their surveillance at all levels [5]. At the international level, there are no dedicated systems to govern official reporting of zoonoses; instead, they are partially covered by separate sets of regulations and requirements for the animal and human sectors. In the human health sector, the IHR provide a legislative framework that formalizes the human disease reporting responsibilities of national governments, including minimum requirements for developing and maintaining core capacities for detecting and responding to emerging threats and a decision support tool designed to help in the identification of public health emergencies of international concern (PHEIC) [13,36,37]. In the animal sector, the closest equivalent to the IHR is the Terrestrial Animal Health Code of the OIE [38], which requires veterinary services in participating states to carry out monitoring and surveillance, and to report animal disease outbreaks to the OIE, particularly of listed notifiable diseases. The OIE Performance of Veterinary Services (PVS) tool is designed to enable the evaluation of the capacity of veterinary services to meet these requirements. Although there are reports of a positive influence of the PVS systems upon reporting [7], these assessments are voluntary guidelines only and the OIE has no capacity to respond to an outbreak without official notification from a member state.

These regulations are designed to encourage reporting of disease outbreaks to the international community. However, they do little to address the significant barriers that act as strong disincentives for a country to report a disease outbreak. Principal among these are the economic and social consequences of reporting outbreaks, which can be extremely severe in terms of imposition of trade embargoes, loss of income from tourism and overall impact upon international reputation [36,39]. The costs of recent zoonoses outbreaks have been estimated at $400 million for Nipah virus in Malaysia, $11 000 million for BSE in the UK and $50–120 000 million for severe acute respiratory syndrome (SARS) globally, largely as a result of losses through animal destruction, trade and tourism [7].

### (c) Barriers to disease reporting

#### (i) Lack of tangible benefits at grassroots level

Possibly the greatest barrier to reporting, particularly within resource-poor systems, has been that efforts to submit diagnostic samples or disease reports often do not result in any feedback or beneficial response to mitigate disease problems for those affected. Few developing countries have systems in place to respond to case-reporting of zoonotic diseases through implementation of disease control measures. The chronic lack of response (or capacity to respond) [40] is disempowering and de-motivating at the grassroots level for healthcare and veterinary workers alike. This problem is further compounded by the potential negative consequences of making a report. Collecting diagnostic samples (e.g. wildlife post-mortem sampling) not only involves time and effort, but can also be unpleasant, arduous and sometimes hazardous. Animal owners and health workers may be unwilling to report illness and initiate a process of investigation that may have severe social and economic implications for both themselves and their neighbours, for example, through trade and movement restrictions or the destruction of animals [2,7]. The absence of direct benefits, combined with the costs of making a report, make it understandable that individuals often choose not to report outbreaks. Addressing these issues remains a high priority. People reporting in the field need to be genuinely integrated as key partners within international surveillance systems and empowered within effective local networks.

#### (ii) Lack of capacity to enforce regulations

In most, if not all, countries, regulations exist that describe legal requirements for both the animal and human health sectors to alert the relevant authorities as and when they become aware of a number of notifiable diseases. The OIE and WHO publish guidelines that describe which diseases are considered notifiable [13,38]. These guidelines list named pathogens that should be reported, and also now define more flexible requirements for reporting of unusual/emerging disease events (e.g. WHO criteria for defining PHEIC and OIE criteria for listing diseases with zoonotic potential and emerging diseases specifically). Within individual countries though, lists of reportable diseases vary according to national priorities. Furthermore, across much of the developing world, and most critically at the local level, the data and infrastructures required to detect non-reporting and to enforce punitive measures are simply not available [7]. In these situations, attempts to impose sanctions for non-reporting on the local scale are unlikely to succeed and will instead further damage relations with animal owners and reduce their engagement as partners within health systems. A more positive approach could be taken to promote the value of reporting, building on small improvements and rewarding change.

#### (iii) Poor communication between institutions at national level

Surveillance is ultimately a matter of communication between stakeholders. It is well-recognized that timely reporting of zoonotic diseases is often hampered by the institutional separation that exists between human and animal health disciplines [2], and an examination of communication between these sectors in the United States identified technological barriers and issues of data sensitivity and trust as barriers to further integration [41]. This illustrates the need to address both the practical elements of communications technology as well as the human element. Individuals in both sectors need to collaborate and trust one another to handle sensitive data.

Within veterinary sectors and increasingly within medical sectors, reporting networks can also be adversely influenced by the separation between private and public sectors and service providers. In many African countries, for example, the policy framework enables private animal health service providers (PAHSPs) to deliver private clinical services, while district veterinary officers (DVOs) are responsible for regulatory and quality control functions. This division often results in DVOs being one step removed from livestock keepers and reliant on PAHSPs for information on any disease outbreak events. There can be significant barriers to this information flow, particularly when relationships between DVOs and PAHSPs are strained by service provision disputes (often relating to poorly applied legislative frameworks), the lack of sanitary mandates and limited recognition and support for frontline personnel by the higher public sector authorities. These problems are not unique to developing country settings. A report on the response to the West Nile virus outbreaks in the USA indicates that better communications between a range of stakeholders, such as wildlife officials, zoo officials, animal health officials, public health agencies and frontline community physicians, would be helpful in tackling future outbreaks [42].

#### (iv) Regulations and rumours

Recognizing that there can be strong disincentives for national governments to report disease outbreaks, the revised IHR now include new powers for the WHO to initiate a response to a PHEIC without official notification from the nation state in which it is detected [2,36]. The move towards the use of such ‘rumours’ for detecting emerging disease threats has been enabled by the recent advances in web-based technologies, and their importance is demonstrated by the fact that the majority of recent outbreak investigations by WHO were initially prompted by unofficial reports [23]. Rapid assessment and response to rumours can provide important health benefits, even in the event of ‘false positives’ (i.e. disease events that turn out to be neither novel, emerging nor of international concern, but nonetheless cause morbidity or mortality). However, reliance upon rumour-based reporting also has potential negative consequences [39]. Rumours do not provide the detailed epidemiological information required to respond as efficiently as possible in an outbreak situation [2]. False positives may divert resources inappropriately and, where countries do not have an adequate surveillance capacity, inaccurate reports or rumours can rapidly lead to social disruption and unwarranted sanctions [39]. These situations apply principally in developing countries that are most susceptible to disease outbreaks, have the least capacity to detect or report them, and are also the least able to withstand the harsh consequences when sanctions are imposed [39]. There is a risk, therefore, of perpetuating a lack of transparency and trust within the global reporting system, while simultaneously failing to enhance the rapid identification of outbreaks.

## 3. Cross-cutting solutions

### (a) Focusing on endemic zoonoses surveillance

Broadening the scope of international surveillance efforts to include both endemic and emerging zoonoses has the potential to address some major existing constraints, by empowering key stakeholders and enhancing core surveillance capacities. In developing surveillance systems for emerging pathogens, there is a temptation to build new systems designed to help the global community detect and respond to these as yet unknown threats. However, there are significant challenges and risks associated with this strategy. First, it is not yet clear how best to build surveillance systems for unknown pathogens and second there are risks that the creation of ‘new’ systems will distort funding priorities and divert much needed resources away from the considerable current infectious disease challenges [43]. By focusing on endemic zoonoses, many of the risks of an ‘emerging only’ approach can be avoided while also helping to tackle an existing public health burden and achieving greater surveillance system sustainability.

To achieve this, it is important to focus efforts and investments on building core capacities that are common to many surveillance systems and inherently adaptable. The rationale behind this approach has been described previously for pandemic influenza preparedness in Africa [43] and more generally for the WHO Africa region Integrated Disease Surveillance and Response (IDSR) strategy [44]. As infectious disease threats have changed, several interdisciplinary networks that were first established to react to specific pathogen threats have subsequently been adapted and used for additional surveillance and response activities not within their original remit. The global network of laboratories established through the Global Polio Eradication Initiative, for example, have since expanded their scope to cover a range of other emerging pathogens, including the haemorrhagic fevers, Japanese encephalitis and SARS, and this network has also contributed resources to national responses to H5N1 influenza [11]. Similarly, efforts are being made to maintain and extend the international collaborations that fostered communication between organizations such as the FAO, World Bank, OIE, WHO and UNICEF during the spread of H5N1 to create a framework that can help reduce a range of disease risks at the animal–human interface [6,7]. These decisions to build on existing systems are demonstrations of the importance of core capacities, such as well-trained personnel and good working relationships, and of the degree to which many key surveillance capacities are transferable across pathogens.

Investment in the surveillance of endemic zoonotic pathogens provides a mechanism for building exactly the core capacities that are likely to enable the detection of emerging infections. In both cases, early detection and crucially early response to a disease outbreak are dependent upon: (i) the awareness of the need for reporting among people involved in the outbreak; (ii) the existence of communication systems through which information can be rapidly reported throughout both animal and human health sectors; (iii) the ability to test and characterize the pathogen involved; and (iv) the existence of trained personnel who can investigate and respond rapidly to the disease event.

There are also added benefits to this kind of approach. In contrast to emerging pathogens, many of the endemic zoonoses have been described as ‘low hanging fruit’ for disease control investments [45], with control tools available and cost-effective strategies evaluated [3]. Endemic zoonoses impose considerable human and animal health burdens [5], and successes in tackling these would be of benefit in their own right. At the same time, surveillance of endemic zoonoses would enable the collection of the baseline surveillance data that are particularly lacking for many developing regions and are crucial in detecting and identifying unusual disease events [42].

Focusing upon developing countries also has benefits. The developing world has been identified as a weak spot in current surveillance capacity; these are the areas where diseases are most likely to emerge and also the communities in which the neglected endemic zoonoses currently impose the greatest burdens. Countries are more likely to invest in surveillance for diseases they can control [46], and the implementation of an effective response to reporting of endemic disease not only reduces the burden of disease in impoverished communities, but also is likely to foster a culture of reporting at grassroots levels. An approach that focuses on diseases that matter to local communities, and on strengthening systems of effective disease control at the local level, provides an opportunity to engage and empower the very people that are relied upon to detect and prevent emerging zoonoses and thus to enhance the sustainability of the surveillance systems. Community-directed schemes for the treatment of onchocerciasis in Africa illustrate the potential of this kind of approach. Schemes to distribute ivermectin treatment for onchocerciasis have strengthened primary healthcare through capacity-building and mobilization of resources while building community confidence in the health system and enhancing relationships between communities and healthworkers [47].

Finally, investments in tackling endemic zoonoses will undoubtedly contribute to building effective communications between institutions, enhancing trust and increasing operational efficiency of outbreak response across human and animal health sectors. Building on small successes can pave the way for more ambitious global goals of preventing emerging pandemics.

### (b) Identifying appropriate incentives for reporting

The appropriate benefits associated with participation in surveillance activities will vary according to the stakeholders involved, but may include meeting performance contracts, remuneration, capacity-building, career advancement, satisfaction and/or reinforcement of social standing (such as by triggering an effective response). Establishment of a global fund to financially compensate countries that report outbreaks and provide assistance at the time of outbreak reporting has been proposed [7,46].

For individual farmers, incentives for reporting may include eligibility for quality certification schemes or compensation schemes in the event of an outbreak in which livestock are compulsorily slaughtered, such as those implemented following the international spread of H5N1 [7]. Although feasible in some wealthier countries, the use of compensation schemes may prove challenging in more resource-limited settings, because a pre-existing record of animal ownership is required and funds need to be readily available to provide timely compensation.

Incentives also need to be considered for reporting of data that reside largely in another sector, e.g. disease information held by private veterinarians. Initiatives designed to build surveillance activities into continuing professional development schemes can provide non-financial incentives, whereby points could be earned in return for undertaking surveillance-related activities focused towards enhanced disease recognition, reporting and information management skills or training of other staff to do so.

Incentives to reporting often focus on tangible financial benefits, but the greatest incentive may be the provision of simple responses, including acknowledgement of a report, feedback of diagnostic test results and advice on management of the disease problem. The provision of relevant and comprehensive information to communities enhances their participation in disease control programmes [47]. Indeed, this is where the benefits of mobile phone technologies become particularly apparent. Well-designed mobile-phone-based systems provide a direct means of communication between sectors, across hierarchies and to at-risk populations, and could therefore greatly empower grassroots surveillance. Our experiences in Tanzania indicate that even simple schemes that involve timely feedback of diagnostic data to livestock-keepers and provision of mobile phone helplines have enormous potential for improving communication links between livestock-keepers and veterinary officers and for supporting the critical first step in the surveillance pathway.

## 4. Conclusions

Multiple factors contribute to the underreporting of zoonoses, particularly in developing countries. Given the complexity of these interacting factors, it is perhaps not surprising that efforts to enhance surveillance have tended to focus on more ‘tangible’ elements, such as laboratory diagnostic infrastructure and communications technology, rather than human interactions, motivation and behaviour. We argue that future investments should build upon a much greater understanding of why individuals choose to act and report disease, rather than focusing exclusively on the technology of the tools used. Effective surveillance on the global scale is only feasible if individuals want to contribute and are not disadvantaged by reporting. Future priorities therefore need to include social science research to understand current barriers to the inclusion of participants, interactions and communication flows within social networks, and the potential value of novel data collection approaches to empower networks of stakeholders to contribute to surveillance [48].

The potential exists for tension between the surveillance needs and motivations of the international community, concerned principally at the global level with the detection of emerging diseases, and developing countries for which endemic diseases pose a greater threat and for which there are few (if any) resources available for either surveillance or response. In the developing world, the current regulatory frameworks, potentially enormous economic costs associated with reporting outbreaks and limited capacity to respond effectively do little to encourage investment in effective disease detection and reporting. Additional investments need to make the best possible use of existing surveillance capacities and focus on building national capacity in a way that ‘works’ for developing countries, meeting their needs, as well as those of the wider global community.

In the developing world, where investment in surveillance has been limited, there is a need to create a ‘culture of surveillance’ [27] and we argue that this is more likely to be achieved by investing in approaches that incorporate meaningful responses that are beneficial to the local communities involved. Targeting surveillance investments at existing zoonotic pathogens would provide a practical solution that not only benefits impoverished communities, but also addresses many of the constraints and barriers to global emerging zoonoses surveillance. Indeed, surveillance and response systems based around interventions that are useful for communities in the immediate future are more likely to be sustainable and can provide the basis for the addition of other surveillance elements and the development of more generic longer-term capacities [46,47]. By investing in surveillance systems that help to control endemic zoonoses, progress can be made towards tackling some immediate health and development problems, demonstrating the practical benefits of surveillance and simultaneously addressing gaps in the capacity of the global surveillance system to respond to future emerging disease threats.
